# Lung Tumor Image Segmentation from Computer Tomography Images Using MobileNetV2 and Transfer Learning

**DOI:** 10.3390/bioengineering10080981

**Published:** 2023-08-20

**Authors:** Zainab Riaz, Bangul Khan, Saad Abdullah, Samiullah Khan, Md Shohidul Islam

**Affiliations:** 1 Hong Kong Center for Cerebro-Cardiovascular Health Engineering (COCHE), Hong Kong SAR, China; zriaz@hkcoche.org (Z.R.); bangukhan2-c@my.cityu.edu.hk (B.K.); mdsislam@hkcoche.org (M.S.I.); 2 Department of Biomedical Engineering, City University Hongkong, Hong Kong SAR, China; 3 Division of Intelligent Future Technologies, School of Innovation, Design and Engineering, Mälardalen University, P.O. Box 883, 721 23 Västerås, Sweden; 4 Center for Eye & Vision Research, 17W Science Park, Hong Kong SAR, China; samikhan@cevr.hk

**Keywords:** deep learning, medical imaging, CT, UNET, MobileNetV2, lung cancer, pulmonary nodule

## Abstract

Background: Lung cancer is one of the most fatal cancers worldwide, and malignant tumors are characterized by the growth of abnormal cells in the tissues of lungs. Usually, symptoms of lung cancer do not appear until it is already at an advanced stage. The proper segmentation of cancerous lesions in CT images is the primary method of detection towards achieving a completely automated diagnostic system. Method: In this work, we developed an improved hybrid neural network via the fusion of two architectures, MobileNetV2 and UNET, for the semantic segmentation of malignant lung tumors from CT images. The transfer learning technique was employed and the pre-trained MobileNetV2 was utilized as an encoder of a conventional UNET model for feature extraction. The proposed network is an efficient segmentation approach that performs lightweight filtering to reduce computation and pointwise convolution for building more features. Skip connections were established with the Relu activation function for improving model convergence to connect the encoder layers of MobileNetv2 to decoder layers in UNET that allow the concatenation of feature maps with different resolutions from the encoder to decoder. Furthermore, the model was trained and fine-tuned on the training dataset acquired from the Medical Segmentation Decathlon (MSD) 2018 Challenge. Results: The proposed network was tested and evaluated on 25% of the dataset obtained from the MSD, and it achieved a dice score of 0.8793, recall of 0.8602 and precision of 0.93. It is pertinent to mention that our technique outperforms the current available networks, which have several phases of training and testing.

## 1. Introduction

Computer tomography (CT) is considered one of the best imaging modalities and has become the standard modality for analyzing and assessing tumors in lungs. The accurate segmentation of cancerous nodules from CT scan images is very important, as it provides necessary information that strongly associates with the early diagnosis of lung cancer and enhances the possibility of patients’ survival [1]. Lung cancer is one of the most fatal cancers worldwide, and malignant tumors are characterized by the growth of abnormal cells in the tissues of lungs [2,3]. Usually, symptoms of lung cancer do not appear until it is already at an advanced stage [4]. A timely diagnosis of malignant lung tumor sub-regions becomes essential for the effective treatment of patients. The Medical Segmentation Decathlon (MSD) is a platform used to analyze and evaluate the development of deep learning models for generalizable 3D semantic segmentation. It provides 3D CT scan images of lung cancer and corresponding annotated ground truths publicly available for the evaluation of models’ robustness. The given CT scans are used for training as well as validating the developed model for the segmentation task.

Qureshi et al. [5] reviewed the semantic-based segmentation methods, existing challenges and their emerging trends. The authors of that study offer insights into the development of machine learning and deep learning mechanisms, along with their strengths and weaknesses. The paper provides a comprehensive overview of recent advancements in semantic segmentation techniques; additionally, it presents a thorough investigation into the effectiveness of different architectures for medical image segmentation. Moreover, it helped the research community by highlighting the benefits, existing challenges and potential future directions.

Traditional methods generally demand handcrafted features, for instance, pixel thresholding, voxel clustering and morphological features [6]. These approaches to medical image segmentation also revolve around edge detection, active contours and template matching techniques [7]. Therefore, deep-learning-based classifiers (DLCs) have changed the research objectives from traditional image processing techniques for feature engineering to network architecture design for obtaining high accuracy. Moreover, transfer learning [8] has established the most practical paradigms in the field of semantic segmentation [9] and image classification [10]. It is a way of utilizing knowledge acquired from a source domain while solving one supervised learning task and employing it to another related target domain. A. A. Mukhlif et al. [11] discussed the significance of accurately segmenting and evaluating the region of interest in medical imaging for disease screening and decision making. The research specifically explored the lung section segmentation from chest X-ray images, training the UNET with one-fold and two-fold training processes. The investigation concluded that the proposed approach achieved superior results in the two-fold training compared to other methods considered in this study. In another study, the authors [9] highlighted the limitation of a CNN to efficiently handle irregular image orientations. To address this problem, a new hybrid deep learning architecture known as the STNCNN was proposed by integrating the space transformer network (STN) with a CNN. The developed model was implemented on a dataset from the Kaggle repository and achieved promising accuracy, outperforming vanilla grey, vanilla RGB and the hybrid CNN.

Due to the heterogeneity of tumors in terms of size, shape and appearance, tumor detection remains a challenge. The automated segmentation of lung tumors from CT scan images can assist medical practitioners in providing an early diagnosis for the further monitoring of disease progression. Classical methods of automatic tumor segmentation mainly depend on feature engineering, which requires the extraction of features from input images for further training of the classifier [10]. However, U-NET, a convolutional neural network, set a new benchmark in biomedical image segmentation and is considered as one of the most advanced techniques for the accurate pattern classification of tumors, as it automatically learns the relevant features [12]. Z Kong et al. [11] presented the hybrid model of MobileNetv2 and UNET for the precise segmentation of liver regions from a liver CT scan dataset. The approach involved introducing random noise to the generator’s input and replacing the fully connected layer with a probability matrix to enhance the discriminator’s sensitivity. The proposed algorithm achieved a dice similarity coefficient of 88.7, surpassing the performance of the standard UNET algorithm.

In this work, we present a deep-learning-based architecture for the semantic segmentation of malignant lung tumors from computed tomographic (CT) images. In our proposed technique, we made the following contributions:

- We utilized a pre-trained MobileNetV2, retaining the convolutional layers, as the encoder of the classical UNET for generating more stable segmentation maps. The decoder part consists of up-sample layers and convolutional layers that recover the spatial resolution and refine the segmentation results.
- Skip connections were established with the Relu activation function for improving the model convergence to connect the encoder layers of MobileNetV2 to the decoder layers in UNet, which allows the concatenation of feature maps with different resolutions from the encoder to decoder. Thus, the decoder leverages both low-level and high-level features for accurate segmentation.
- Finally, we added a 1 × 1 convolution layer at the end of the decoder to reduce the number of channels and to obtain the number of output classes, such as tumor and background.
- The devised network was further trained and fine-tuned with optimized hyper-parameters on the training dataset obtained from the Medical Segmentation Decathlon (MSD) 2018 Challenge.
- The results indicate that the proposed approach is robust and significantly improved the segmentation accuracy.

The rest of the paper is organized as follows: Section 2 of this paper indicates the literature covering the machine learning and deep learning techniques used in this domain. Section 3 elaborates a detailed explanation of the proposed methods and Section 4 focuses on the results and discussion, in which the obtained results from the suggested algorithm are discussed and presented.

## 2. Background

The precise assessment of a lung tumor is essential to scrutinize its malignancy and the probability of lung cancer. Wang et al. [13] proposed a support vector machine (SVM) based on the three-dimensional matrix pattern method to avoid the loss of local and structural information. The three-dimensional volume of tumors took the whole region of interest (ROI) for analysis and fed it as an input image for the training of the algorithm, and the model was not able to classify between benign and malignant tumor. However, the lung parenchyma segmentation technique using the fast marching method was adopted in [14] to extract candidate nodules from segmented lung parenchyma. Afterwards, a random forest (RF) algorithm was employed for the classification between benign and malignant tumors.

Mukhlif et al. [15] highlighted the need for smart systems to aid clinicians in the early detection of breast cancer, where the authors aimed to address the non-medical nature of ImageNet features by incorporating unclassified medical images of the same disease to mitigate the reliance on ImageNet. Therefore, the proposed approach employed a modified Xception model to classify histological images of breast cancer into four categories, and achieved high performance compared to previous studies in this field. On the other hand, S Lu et al. [16] aimed to develop a system for automatically identifying COVID-19 in chest CT images using artificial intelligence. The researchers utilized transfer learning to obtain image-level representations (ILRs) based on a deep CNN. They proposed a neighboring aware representation (NAR) to capture neighboring relationships between ILR vectors. Based on such representations, they introduced a novel COVID-19 classification architecture known as NAGNN that outperformed the state-of-the-art methods in terms of generalizability.

S M Naqi et al. opted for a strategy of employing multiple ML techniques for the detection of lung cancer and compared the obtained results. Geometric texture and 3D component connectivity was analyzed by novel hybrid 3D nodule detection, and based upon the extracted feature, classification was performed by K-Nearest Neighbors (KNN), SVM and AdaBoost. The evaluation of AdaBoost was performed using a dataset acquired from the Lung Image Database Consortium (LIDC) [17].

W. Choi and T. Choi [18] suggested an automatic approach for the identification of a lung tumor on the basis of a feature descriptor which then differentiated by the 3D shape of the tumor. Multi-scale dot enhancement filtering is a technique utilized for segmenting lung volume. Afterwards, potential nodule candidates were extracted and refined by using an iterative edge elimination algorithm. Finally, an SVM classifier was trained to differentiate nodules and non-nodules. M. Usman et al. [19] devised an approach that consists of two stages: the first stage provides an initial estimation of a tumor by performing patch-wise exploration along the axial axis using an adaptive ROI algorithm. In the second stage, the extracted region is further investigated for the existence of a malignant tumor along the coronal and sagittal axes.

The algorithm proposed by A. Setio et al. [20] was composed of three candidate detectors specially designed for the detection of cancerous lesions to enhance the detection sensitivity of lesions. The nodule candidates were computed and processed by ConvNets by averaging the position of the tumor and its probability. U Kamal et al. proposed the recurrent 3D-DenseUNet, an architecture for the segmentation of the volume of interest from lung CT scans. The suggested approach comprised a 3D encoder block and recurrent block of ConvLSTM layers to bring out fine-grained spatio-temporal details and later reconstruct the volumetric segmentation mask by introducing a 3D decoder block. S Lu et al. [21] proposed a novel method for detecting abnormal brain regions in MRI images using a pre-trained AlexNet model. The authors modified a pre-trained model by adding batch normalization layers and replaced the last layers with an extreme learning machine. Furthermore, the extreme learning machine was optimized utilizing a chaotic bat algorithm to enhance the classification performance, which demonstrated state-of-the-art results in abnormal brain region detection.

Random transformation induces deliberate changes and can be used to create varied images from available images to enhance the size of a dataset for training the classifier. Deep convolutional neural networks (CNNs) have performed exceptionally well on computer vision tasks. Overfitting happens when a network understands a function with high variance. However, data augmentation increases the data size, along with the class-preserving transformation and standards of the training dataset, ultimately strengthening the generalization ability of deep learning models [22].

Tri Dao et al. [23] established a theoretical framework for understanding data augmentation schemes. The Markov process is a general model of augmentation where kernels appear spontaneously in the model. Data augmentations can be approximated by first-order feature averaging and second-order variance regularization components. They also analyzed the methods of augmentation that modify the models’ learning ability. Nonetheless, data augmentation enhances the training dataset size by geometric and color transformations and adversarial training.

### Deep Learning Techniques

Deep learning architectures give exceptional results on tasks of semantic segmentation as compared with classical machine learning and context-based computer vision methods. M. Havaei et al. [24] presented a deep neural networks (DNN) for brain tumor segmentation to fully automate the approach, in which local features and global contextual features were utilized simultaneously to enhance the robustness of the network. The model outperformed on the BRATS dataset compared to state-of-the-art approaches. T. Brosch et al. [25] put forward a novel segmentation framework that relies on deep 3D convolutional encoder networks along with shortcut connections and employed it to segment out the lesions from magnetic resonance images (MRI).

The suggested network mainly comprised two inter-connected pathways, a convolutional path, which ascertains more abstract and prominent image features, and a deconvolution path, which anticipates segmentation at the voxel level. The model was validated on the publicly available MICCAI 2008 dataset with promising results. Xiaomeng Li et al. [26] concentrated on a Hybrid Densely Connected UNET, which was comprised of a 2D DenseUNet for the extraction of features and a 3D counterpart for accumulating volumetric contexts to segment out the liver tumor. Fabian Isensee et al. [27] introduced the robust no-new-Net (nnU-Net) framework, where the Relu activation function is replaced by leaky Relu and instance normalization is used instead of batch normalization. Furthermore, they evaluated the model using the Medical Segmentation Decathlon Challenge (MSD) dataset and achieved the highest mean dice score.

Çiçek et. al. [28] presented an architecture for volumetric segmentation where a network from Ronneberger et al [29]. was extended by replacing all two-dimensional operations with three-dimensional counterparts. The suggested network was trained from scratch and data augmentation schemes were also implemented during training. The performance of the network was tested on the complex 3D structure of Xenopus Kidney and accomplished good results. Transfer learning enables the new model to benefit from previous knowledge by leveraging the learned features and representations; therefore, A A Mukhlif et al. [30] discussed the applications of transfer learning in various domains, particularly image processing and interpretation. They also revealed the prevalent use of pre-trained models from the ImageNet dataset in applications such as skin cancer, breast cancer and diabetic retinopathy classification. Along with that, the authors further investigated the problems in melanoma and breast cancer datasets, and potential solutions were suggested. In another study, A A Mukhlif et al. [31] discussed the limitations of transfer learning in the medical domain due to the mismatch between the source and target problem. To address this issue, the study proposed a novel approach known as dual transfer learning (DTL) that focused on the convergence of patterns between two domains. The proposed approach employed four pre-trained models utilizing two datasets: skin cancer images and breast cancer images. The final layers of the models were fine-tuned on enough unclassified images of the same disease and a small number of classified images from the target task. The experimental results demonstrated that the proposed approach improved the performance of all models.

## 3. Materials and Methods

### 3.1. Dataset

The dataset for training, validation and evaluation of the proposed algorithm was obtained from the Medical Segmentation Decathlon Challenge (MSD). The 3-dimensional CT image dataset, acquired from The Cancer Imaging Archive (TCIA), was made available to the public through the Medical Segmentation Decathlon Challenge (MSD). Briefly, 96 preoperative thin-section CT images were obtained with the following parameters: automatic tube current modulation range, 100–700 mA; helical pitch, 0.9–1.0; tube rotation speed, 0.5 s; section thickness, <1.5 mm; 120 kVp; and a sharp reconstruction kernel [32]. The training set used here comprises 64 heterogeneous CT images with accurately annotated ground truths, which we further split into training, validation and test sets to analyze the validity of the proposed architecture.

**Figure 1:**
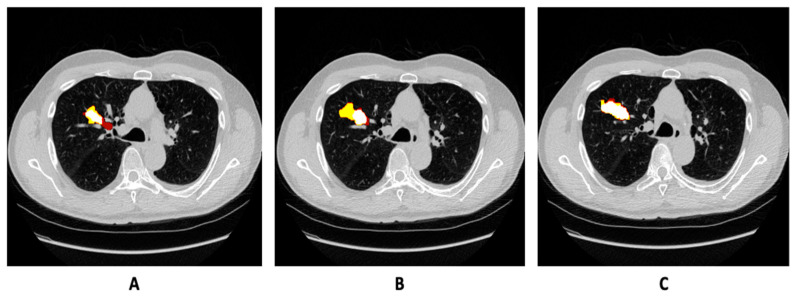
(**A**–**C**) Normalized image slices of CT scan of same patient with growing tumor in MSD-2018 training set, along with annotation overlaid on the image.

Each CT scan volume has a dimension of 512 × 512 × X, where X denotes the variability in voxel size of each CT scan. From these CT volumes, the segmentation of the tumor sub-region was performed. Therefore, the dataset was processed to overcome the inconsistency of the voxel of each 3D scan by splitting into 2D images, wherein lung nodules also had huge variations in tumor size and morphological characteristics. Different 2D slices from 3D CT scans and their corresponding ground truths are shown in Figure 1 as example images from the training set. We did not adopt datasets other than the mentioned dataset in the experiments, and precise segmentation results from the suggested model were then compared to the existing state-of-the-art networks. We provide a further detailed explanation on the methods utilized to process the dataset in the following section.

### 3.2. Methodology

In this section, we begin by describing the architecture that we employed. MobileNetV2 is usually adapted for resource-constrained environments to accurately solve the problem of semantic segmentation and has the advantage of improving segmentation results. We propose a computationally lightweight network with fewer trainable parameters, and it achieves a perfect balance between performance results and implementation efficiency. A 2D image containing the nodule was provided as an input to detect the presence of lesions using an algorithm. The output of the network was a segmentation map, from which a dice score coefficient was calculated. We provide further details on the pipeline that has several phases in the subsequent sections.

#### Preprocessing

Image normalization: We converted the 3D computed tomographic (CT) images to 2D and resized them to 256 × 256 to reduce the size of the CT slices owing to memory consideration. Furthermore, the images were normalized to minimize poor contrast issues before feeding them into the model for training. The following min–max approach rescaled the feature in the range of 0 and 1.

(1) $$I_{normalized}=\frac{I-I_{min}}{I_{max}-I_{min}}$$

**Figure 2:**
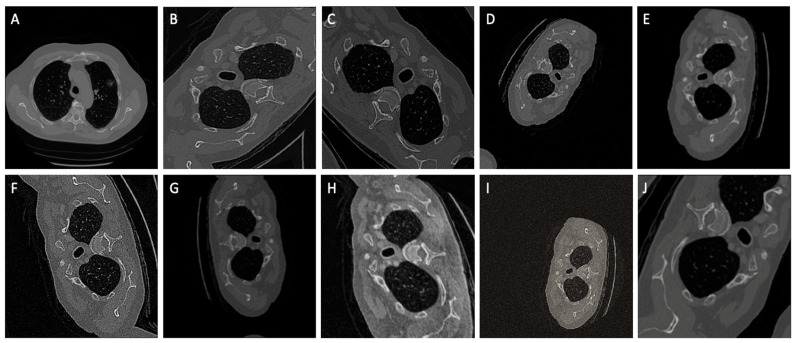
A comprehensive set of ten random augmentations, denoted as (**A**–**J**), that were strategically employed to enhance the dataset size and elevate the model’s generalizability during the training phase.

Data Augmentation: When training the neural network with limited training data, special attention must be paid to minimize overfitting. Augmentations induce deliberate changes and hence can be used to create varied images from the available image dataset. Greater variation in training data ensures model generalization. Images are randomly augmented, which reduces the possibility of modeling to learn inherent patterns in data. Augmentations as illustrated in Figure 2. such as CLAHE, rotate, blur, random contrast, random sized crop and Gaussian blur are applied on data during runtime to circumvent overfitting and to enhance the segmentation accuracy.

### 3.3. Network Architecture

The encoder–decoder-based architecture is a classical U-NET with MobileNetV2 as the pre-trained encoder; however, U-NET is a fundamental convolutional neural network (CNN), initially developed by Olaf Ronneberger et al. [29] for biomedical image analysis, and has received appreciation in the medical imaging community. On the other side, MobileNetV2 [33,34] introduced lightweight convolutions in the encoder part of the network and achieved highly accurate results with much fewer parameters. Additionally, skip connections were established with the Relu activation function to increase the model’s convergence to connect encoder layers to decoder layers, which further allowed the concatenation of feature maps with different spatial resolutions. The encoder takes an image as the input of the model and extracts necessary features and relevant information, whereas the decoder learns to generate the corresponding predictions (probability maps). Furthermore, skip connections in the down-sampling path are concatenated with feature maps in the up-sampling path to provide local information to global information.

### 3.4. Model Training

**Figure 3:**
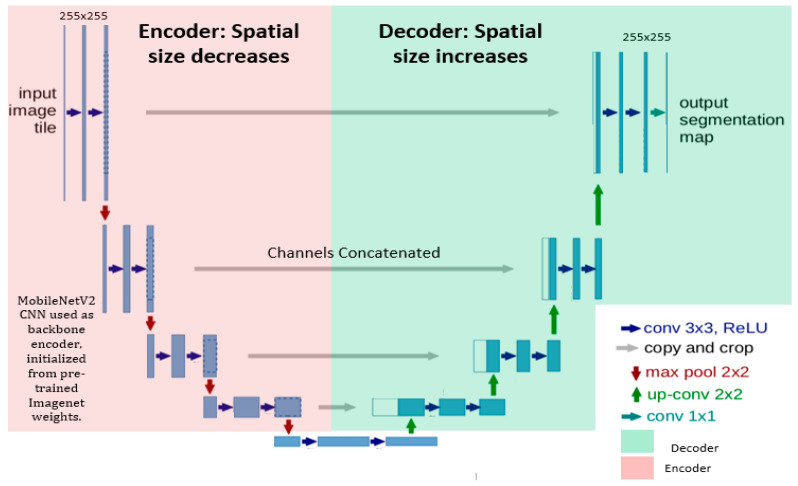
A structural visualization of the network architecture, where the encoder exhibited on the left side is MobileNetV2 and the U-NET decoder is shown on the right side. Input of patch size 256 × 256 was given into the model. Convolutional units were used with batch normalization and Relu function activations. Up-sampling along with concatenated feature channels were employed to obtain the output of the same spatial size as that of the input.

We trained the model for 90 epochs with a patch size of 256 × 256 and batch size of 8. Fine-tuned hyperparameters are demonstrated in Table 1. We used dice loss, as it performs better and gives more preference to true positives compared to Jaccard loss and binary cross-entropy loss. Binary cross-entropy loss saturates too quicky owing to large black pixel areas in medical images. Pre-trained weights were initialized and trained on the large ImageNet dataset; thus, the hybrid model leveraged the learned generic image features. The learning rate is reduced by a factor of 0.01 if the validation loss does not decrease continuously for four epochs. Moreover, training would be stopped if the validation dice loss remained unchanged up to 10 epochs. Along with that, an Adam optimizer was used to update the model’s weights to enhance model’s learnability. Moreover, a shortcut connection was incorporated to enable the flow of the gradients, improve feature reuse and enhance network performance Moreover, the overview of the model layout is highlighted in Figure 3.

### 3.5. Evaluation Parameters

We used the following performance evaluation matrices to measure the robustness of the classifier.

#### 3.5.1. Dice similarity Coefficient (DSC)

The DSC is the degree of overlap of the predicted segmentation with reference segmentation [20,22]. The DSC (shown in Equation (2)) value range is [0, 1], where 1 and 0 indicate perfect agreement and no overlap, respectively. The formula comprehension of the dice coefficient is given below.

(2) $$DSC=\frac{2TP}{2TP+FP+FN}$$

#### 3.5.2. Dice Loss (DL)

The loss function calculates the degree of inconsistency between the predicted value of the model and the ground truth value. We employed the simple dice coefficient loss function that is the negation of the dice score coefficient, used in this experiment to determine the measure of intersection between regions.

(3) $$Loss=L_{dice}=1-DSC\;$$

#### 3.5.3. Recall and Precision

Recall and precision together were the measures used to evaluate the effectiveness of the classification model. Recall is basically the proportion of correct positive classification from the cases that are positive. True positives are the data points identified as positive by the classifier that are correct. And false negatives are data points the model classifies as negatives that are positives and are incorrect.

(4) $$Recall=\frac{True\;Positive}{Predicted\;Results}=\frac{nTP}{nTP+nFN}$$

Precision is the ratio between the true positives and all the positives, and also expounds the proportion of the relevant instances among all retrieved instances.

(5) $$Precision=\frac{True\;Positive}{Actual\;Results}=\frac{nTP}{nTP+nFP}\;$$

## 4. Results

**Figure 4:**
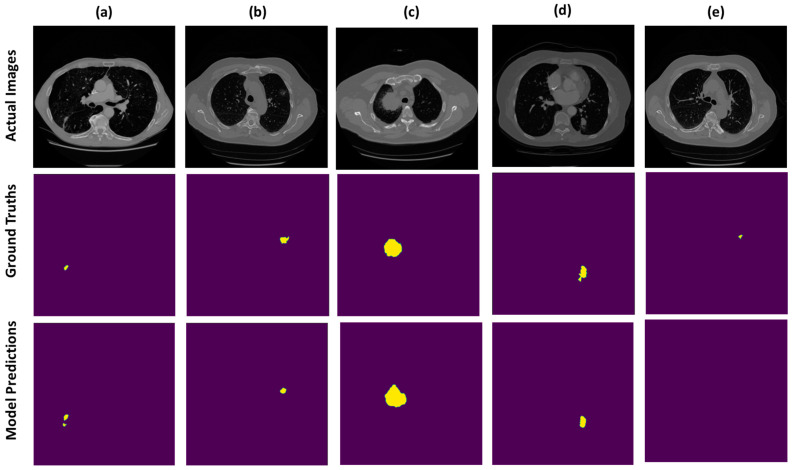
Example CT scans of different patients are exhibited in the form of rows. First row indicates the actual images, middle row is the visualization of true labels and last row is the segmentation predictions, wherein most of the prediction results are correctly segmented as visualized in (**a**–**d**) and very few of them are omitted by the model as depicted in (**e**).

**Figure 5:**
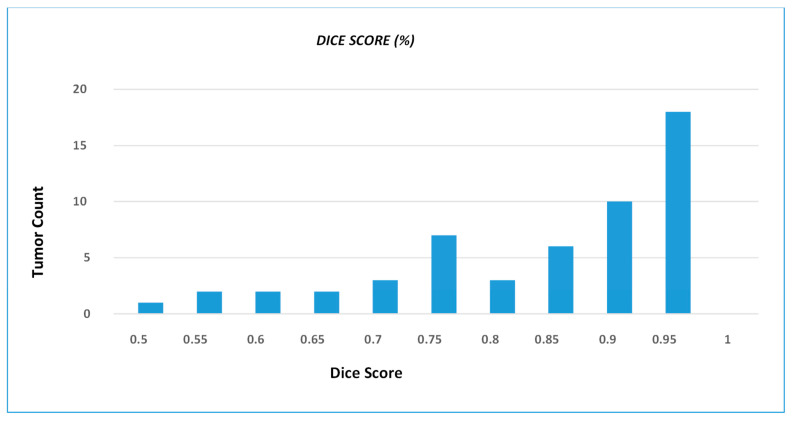
DSC distribution of the test dataset, wherein histogram shows number of tumors that achieve particular dice score coefficient. The histogram graphically illustrates the frequency with which different tumor instances achieve particular DSC values.

We present the prediction results from our devised segmentation model, evaluated using the MSD-2018 lung tumor segmentation dataset. We used U-NET architecture by integrating the down-sampling path of the U-NET with a pre-trained MobileNetV2 encoder that was trained on a large ImageNet dataset. The prediction maps generated from the proposed network are shown in Figure 4. The dice score achieved by the network is 0.8793 and the recall and precision of model are 0.8602 and 0.9322, respectively. Moreover, the distribution of the dice score coefficient of each patient is illustrated in a histogram (shown in Figure 5), and the average dice score that we achieved is 0.8793. Therefore, the proposed method trained the deep neural network and validated it with the Medical Segmentation Decathlon (MSD) lung CT scan dataset, showing competitive results as compared with the state-of-the-art methods.

### Result Comparison with Existing Methods

In this section, we present the prediction results from our segmentation model evaluated using the MSD-2018 lung tumor segmentation dataset and compare our results with various state-of-the-art deep learning methods (shown in Table 2) that are validated on a lung CT scan dataset. Table 2 depicts the results of the mentioned techniques in terms of the dice score coefficient (DSC). These approaches utilized complex pipelines of training and achieved comparable results, whereas our framework is computationally light and gives better accuracy and performed reasonably well in capturing the whole nodule shape. We confirmed the effectiveness and efficiency of our fine-tuned model with extensive experiments, and it can be applied to other medical segmentation tasks with required modifications suited to the task.

## 5. Conclusions and Discussion

This study has addressed the critical challenge of lung cancer detection and diagnosis through the development of an innovative hybrid neural network. By using the strengths of MobileNetV2 and UNET architectures, we have achieved remarkable strides in the semantic segmentation of malignant lung tumors from CT images.

The urgency of early lung cancer detection cannot be overstated, as symptoms typically manifest at advanced stages. Our proposed network’s ability to accurately segment cancerous lesions within CT images marks a pivotal advancement toward a comprehensive automated diagnostic system. This initial step is essential for enhancing patient outcomes through timely interventions. The adoption of transfer learning, specifically integrating the pre-trained MobileNetV2 as the encoder, underscores the potency of leveraging existing knowledge to expedite model training and enhance feature extraction. This integration not only bolsters the model’s efficiency but also capitalizes on the MobileNetV2’s lightweight characteristics, optimizing computational resources.

The model’s segmentation efficiency is a standout feature, attributed to its adept utilization of lightweight filtering and pointwise convolutions. This strategic approach streamlines computations without compromising feature richness, which is crucial for accurate segmentation. The incorporation of skip connections, augmented by the Relu activation function, facilitates seamless information flow between encoder and decoder layers. This design innovation contributes to the improved model convergence and overall performance.

Our model’s performance, validated through testing on a subset of the MSD dataset, demonstrated a dice score of 0.8793, recall of 0.8602 and precision of 0.93. These results underscore the effectiveness of our approach, outperforming existing networks that necessitate multiple training and testing phases [41]. Our technique’s ability to achieve superior segmentation accuracy with a single model training phase holds significant promise for expediting the diagnostic process.

The impact of our research extends beyond segmentation accuracy. By enabling the early and precise identification of malignant lung tumors, our methodology has the potential to transform clinical decision making and patient management. Rapid and accurate tumor segmentation aids clinicians in assessing disease progression and tailoring treatment strategies, ultimately enhancing patient care.

## 6. Limitations and Future Prospects

Within the scope of this study, specific limitations warrant consideration. Our proposed segmentation method underwent evaluation solely on the validation set of the challenge. To ascertain its robustness, extending testing to diverse medical image segmentation tasks, independent of the challenge dataset, would be imperative. While post-processing of our segmentation results was not exhaustive, exploring the integration of Conditional Random Fields (CRF) [42] holds potential for enhancing segmentation accuracy. Furthermore, the susceptibility to overfitting, particularly with limited or imbalanced training data, could affect model performance. The augmentation of data during training can mitigate such concerns, preventing the undue memorization of training data.

In the future, comprehensive research endeavors are necessary to forge robust computer-aided detection (CAD) models or optimize existing networks. These advancements stand to empower clinicians in achieving accurate and timely lung tumor detection and quantitative assessment. Importantly, our segmentation approach remains impartial, deriving essential features exclusively from training data without preconceived assumptions about suspicious lesions. This enables its applicability across various 2D pathological segmentation tasks when compatible data are available. Furthermore, our proposed framework exhibits adaptability and can be readily refined. For instance, the integration of a multi-scale Gaussian distribution into CT images could enhance the feature evolution process. In our forthcoming work, we intend to adapt the model architecture to a 3D convolutional neural network to explore its performance in a broader spectrum of medical imaging tasks.

## Figures and Tables

**Table 1:** Hyperparameters used for CNN training.

Name	Value
Input size	255 × 255
Batch size	8
Learning rate	1 × 10^–4^
Epoch Activation head	90 sigmoid
Optimizer	Adam
Loss function	Ldice

**Table 2:** Dice score coefficient (DSC) comparison with different architectures.

Approach	DSC (%)
Central Focused CNN [35]	0.821
Multichannel ROI based on Deep Structured Algorithm [36]	0.7701
Multi-Crop CNN [37]	0.7751
Multi-View Deep CNN [38]	0.7767
Cascaded Dual-Pathway Residual Network [39]	0.8158
Unsupervised Metaheuristic [40]	0.8235
Proposed Method	0.8793

## Data Availability

All data are available in the paper.
